# Exosomes: Novel Biomarkers for Clinical Diagnosis

**DOI:** 10.1155/2015/657086

**Published:** 2015-01-27

**Authors:** Jin Lin, Jing Li, Bo Huang, Jing Liu, Xin Chen, Xi-Min Chen, Yan-Mei Xu, Lin-Feng Huang, Xiao-Zhong Wang

**Affiliations:** ^1^Department of Clinical Laboratory Medicine, The Second Affiliated Hospital of Nanchang University, Nanchang 330006, China; ^2^Department of Clinical Laboratory Medicine, The First Affiliated Hospital of Nanchang University, Nanchang 330006, China

## Abstract

Exosomes are 30–120 nm endocytic membrane-derived vesicles that participate in cell-to-cell communication and protein and RNA delivery. Exosomes harbor a variety of proteins, nucleic acids, and lipids and are present in many and perhaps all bodily fluids. A significant body of literature has demonstrated that molecular constituents of exosomes, especially exosomal proteins and microRNAs (miRNAs), hold great promise as novel biomarkers for clinical diagnosis. In this minireview, we summarize recent advances in the research of exosomal biomarkers and their potential application in clinical diagnostics. We also provide a brief overview of the formation, function, and isolation of exosomes.

## 1. Introduction

Exosomes are small cell-derived vesicles of 30–120 nm that are present in many and perhaps all biological fluids. Exosomes were first discovered in the mid-1980s by the Johnstone group, who found that, in maturing mammalian reticulocytes, the transferrin receptor and some other membrane-associated elements are selectively released in multivesicular body- (MVB-) derived circulating vesicles, which they named exosomes [1–3]. Since Valadi et al. first reported in 2007 that exosomes also contain RNAs [4], the composition and function of exosomes have been intensely investigated. Now we know that exosomes carry various molecular constituents of their cell of origin, including proteins, lipids, mRNAs, and microRNAs (miRNAs). They are released from many cell types, such as dendritic cells (DCs), lymphocytes, platelets, mast cells, epithelial cells, endothelial cells, and neurons, and can be found in most bodily fluids including blood, urine, saliva, amniotic fluid, breast milk, hydrothoracic fluid, and ascitic fluid, as well as in culture medium of most cell types [5]. Exosomes were initially thought to serve simply as “garbage bags” for cells to get rid of unwanted constituents. However, an increasing body of evidence has demonstrated that exosomes play an important role in cell-to-cell communication and influence both physiological and pathological processes. Additionally, molecular constituents in exosomes have been found to be associated with certain diseases and treatment responses, indicating that they may also serve as a diagnostic tool. A PubMed search generated a list of over 1,000 exosome-related articles published in the past 5 years, showing the increasing level of interest in the biomedical research community. In this review, we summarize recent progress in the study of exosomes as novel biomarkers for clinical diagnosis. We also provide a brief overview of the formation, function, and isolation of exosomes.

## 2. Formation and Composition of Exosomes 

MVBs are late endosomes that carry many “intraluminal endosomal vesicles.” Some MVBs are destined for degradation in lysosomes, while other MVBs merge with the cell membrane and release the internal vesicles into the extracellular space. Of the released vehicles, the smaller ones with a diameter of 30–120 nm are called exosomes; and the larger vehicles with a diameter of 120–1000 nm are called microvesicles [6–8]. Exosome formation involves the endosomal sorting complex required for transport (ESCRT), which recognizes ubiquitylated proteins. In addition to ESCRT, other ESCRT-independent mechanisms operate to generate exosomes of certain biochemical compositions [9]. Exosomes isolated by differential ultracentrifugation have a cup-shaped morphology as revealed by electron microscopy imaging [10].

Exosomes have a unique and complex composition. According to Exocarta (Version 4; http://www.exocarta.org), the latest exosome content database, 4,563 proteins, 194 lipids, 1,639 mRNAs, and 764 miRNAs have been identified in exosomes from multiple organisms [11]. The proteins most frequently identified in exosomes are membrane transporters and fusion proteins (e.g., GTPases, annexins, and flotillin), heat shock proteins (e.g., HSC70), tetraspanins (e.g., CD9, CD63, and CD81), MVB biogenesis proteins (e.g., alix and TSG101), and lipid-related proteins and phospholipases [10, 12]. Several proteins are recognized as specific exosomal markers, among which the tetraspanins, CD63 and CD81, are the most commonly used. Exosomes are also rich in lipids, which are predominantly cholesterol, sphingolipids, phospholipids, and bisphosphates. The exosomal lipid composition has been thoroughly analyzed in exosomes secreted from several cell types including DCs and mast cells [13], reticulocytes [14], and B-lymphocytes [15]. Several reports have suggested that certain lipid components of exosomes, such as phosphatidylserine [16] and prostaglandins [17], may play an important role in exosomal functions. The discovery that exosomes also contain mRNAs and miRNAs indicates that exosomes could be a carrier of genetic information. Although the majority of RNAs found in exosomes are degraded RNA fragments with a length of less than 200 nucleotides, some full length RNAs might be present and may be shuttled to a recipient cell via endocytosis and potentially affect protein production in the recipient cell. Meanwhile, exosomal miRNAs have been found to be associated with certain diseases. For instance, several studies have noted that miRNA contents of circulating exosomes are similar to those of their originating cancer cells, suggesting that exosomal miRNAs have potential for cancer diagnostics [18–20]. Also, an increasing number of studies have reported that miRNAs can be detected in exosomes isolated from noninvasively obtained bodily fluids such as saliva [21], showing potential advantages of exosomal miRNAs as novel biomarkers.

## 3. Function of Exosomes

**Figure 1:**
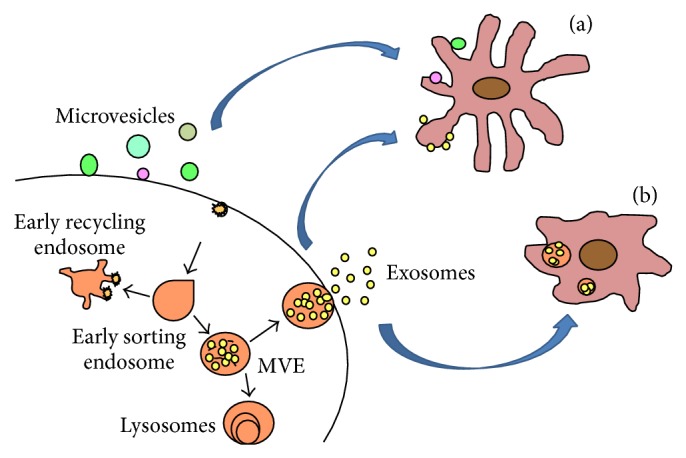
Exosomes are released from host cell and uptook by recipient cells. Exosomes are generated in host cell by merging MVBs with the cell membrane and releasing into the extracellular space. These exosomes can be fused with the plasma membrane (a) or be internalized (b) by recipient cells.

Exosomes can merge with and release their contents into recipient cells (Figure 1). By transferring cellular constituents of proteins, RNAs, and lipids from one cell to another, exosomes play an important role in cell-to-cell communication [22–24]. A substantial body of literature has demonstrated that exosomes exhibit a broad range of functions, depending on their cell or tissue of origin. Particularly, exosomes from certain types of immune cells, such as DCs and B cells, may mediate adaptive immune responses to pathogens and tumors [25]. Tumor cell-derived exosomes play an active role in tumorigenesis, metastasis, and response to therapy through the transfer of oncogenes and onco-miRNAs between cancer cells and between cancer cells and the tumor stroma [26]. Exosomes shed from stimulated blood cells and the vascular endothelium is involved in neurological disorders such as multiple sclerosis, transient ischemic attacks, and antiphospholipid syndrome [27]. Interestingly, a few recent studies have shown that exosomes are also exploited by pathogens such as prions or viruses to transfer molecules of pathogenic origin between host cells and are thereby implicated in viral spread and immune evasion [28, 29]. Furthermore, since the molecular composition of exosomes is reflective of physiological or pathophysiological changes in their cell or tissue of origin, exosomes have significant potential as biomarkers for disease diagnosis.

## 4. Isolation of Exosomes

Owing to their small size and low density, exosomes are usually isolated from bodily fluids and cell culture media by differential ultracentrifugation [44, 45]. Briefly, the collected biofluid is centrifuged at 300 ×g followed by a second centrifugation at 10,000 ×g to remove dead cells and cell debris. The supernatant is collected and subjected to ultracentrifugation at 100,000 ×g for 1 h or more. The pellet containing crude exosomes is subsequently washed with phosphate buffer solution to remove remaining proteins and other contaminants. The sample is subjected to a second ultracentrifugation at 100,000 ×g to yield purified exosomes. Several studies have also shown that exosomes may be isolated with higher purity using ultracentrifugation in a continuous density gradient of sucrose [46, 47].

Ultracentrifugation not only is labor-intensive and time-consuming but also requires expensive laboratory equipment, making it unsuitable for clinical applications. However, several recent technical advantages have made exosome isolation easier and faster and thus more cost-efficient. Cheruvanky et al. [48] and Merchant et al. [49] have successfully used ultrafiltration and microfiltration, respectively, for rapid isolation of urinary exosomes. Exosomes have also been isolated by immunoaffinity capture methods using lectins or antibodies against exosomal markers such as CD63, CD81, EpCAM, or Rab5 [45, 50, 51]. Precipitation followed by centrifugation is another method that has been explored for rapid exosome isolation. Nowadays, exosomes can be isolated in a one-step precipitation procedure using commercial reagents such as ExoQuick (System Biosciences, Mountain View, CA, USA). The Rekker group has demonstrated that ExoQuick is as efficient as ultracentrifugation in isolating serum exosomes for exosomal miRNA profiling and may be more efficient than ultracentrifugation in the context of exosomal RNA analysis [52].

## 5. Exosomes in Diagnostics

Exosomes are shed by cells under both normal and pathological conditions. They carry nucleic acids and proteins from their host cells that are indicative of pathophysiological conditions, and they are widely considered to be crucial for biomarker discovery for clinical diagnostics. For instance, tumor cells release exosomes containing tumor-specific RNAs that can be potentially used for cancer diagnosis. Over the past few years, numerous studies have demonstrated that exosomes contain nucleic acids and proteins implicated in cancer as well as neurodegenerative, metabolic, infectious, and other diseases. Furthermore, exosomes can be isolated from easily attainable biofluids such as blood and urine, making them very attractive targets for diagnostic application. In this review, we briefly summarize the main research advances reported to date in the context of diagnostic applications of exosomes.

### 5.1. Exosomal Proteins as Diagnostic Biomarkers

Exosomes contain diverse types of proteins including common membrane and cytosolic proteins as well as origin-specific subsets of proteins reflective of cell functions and conditions. Recently, an increasing number of exosomal proteins have been found to be potential biomarkers for a variety of diseases including cancer as well as liver and kidney diseases.

Tetraspanins, a family of scaffolding membrane proteins, are highly enriched in exosomes. The exosomal marker CD63 is a member of the tetraspanin family. Logozzi and coworkers reported in 2009 that plasma CD63^+^ exosomes are significantly increased in melanoma patients compared with healthy controls [31]. Most recently in 2013, Yoshioka and coworkers performed a comparative analysis of exosomal protein markers in different human cancer types and found that CD63 is present at higher levels in exosomes derived from malignant cancer cells than those derived from noncancer cells, providing further evidence that exosomal CD63 could be a protein marker for cancer [65]. CD81, another exosomal marker from the tetraspanin family, plays a critical role in hepatitis C attachment and/or cell entry. In addition, Welker and coworkers reported in 2012 that the level of serum exosomal CD81 is elevated in patients with chronic hepatitis C and seems to be associated with inflammation and severity of fibrosis, suggesting that exosomal CD81 may be a potential marker for hepatitis C diagnosis and treatment response [30].

A number of exosomal protein biomarkers have been found to be potentially useful in the diagnosis of central nervous system diseases. In 2008, Skog and coworkers detected glioblastoma-specific epidermal growth factor receptor vIII (EGFRvIII) in serum exosomes isolated from 7 out of 25 glioblastoma patients, indicating that exosomal EGFRvIII may provide diagnostic information for glioblastoma [33]. A year later, in line with Skog's findings, Graner et al. reported that serum exosomes from patients with brain tumors possess EGFR, EGFRvIII, and TGF-beta [66]. It has also been reported that exosomal amyloid peptides accumulate in the brain plaques of Alzheimer's disease (AD) patients [67]; and tau phosphorylated at Thr-181, an established biomarker for AD, is present at elevated levels in exosomes isolated from cerebrospinal fluid specimens of AD patients with mild symptoms [68]. These findings highlight the potential value of exosomes in the early diagnosis of AD, which is very important in sabotaging disease progression but currently difficult to achieve. Studies have also shown that *α*-synuclein, whose aggregation plays a central role in Parkinson's disease pathology, is released in exosomes in an* in vitro* model system of Parkinson's disease [69]; and prion proteins, biomarkers for transmissible spongiform encephalopathies, are packaged into exosomes released from prion-infected neuronal cells [70]. These exosomal proteins may have great potential in clinical diagnostics and should be further explored.

Proteins in urinary exosomes, which are easily attainable by noninvasive means, have also been exploited for potential utility in diagnostics, especially for urinary tract diseases. In 2006, Zhou et al. found that urinary exosomal fetuin-A is increased in intensive care unit patients with acute kidney injury (AKI) compared with patients without AKI [36]. Two years later, the same group reported that activating transcription factor 3 was found in exosomes isolated from patients with AKI but not from patients with chronic kidney disease or controls [37]. The authors thus concluded that measurement of these urinary exosomal proteins might offer diagnostic information for AKI. Urinary exosomal proteins have also been investigated as potential biomarkers for bladder cancer and prostate cancer. In 2008, Smalley et al. compared the protein profile of urinary exosomes between patients with bladder cancer and healthy controls and identified eight urinary exosomal proteins as potential biomarkers for bladder cancer, including five proteins associated with the EGFR pathway, the alpha subunit of Gs protein, resistin, and retinoic acid-induced protein 3 [40]. In 2009, Nilsson and coworkers demonstrated the presence of two known prostate cancer biomarkers, PCA-3 and TMPRSS2:ERG, in exosomes isolated from the urine of prostate cancer patients [41]. In 2012, Chen et al. identified that 24 urinary exosomal proteins were presented at significantly different levels between bladder cancer and hernia (control) patients (*P* < 0.05) [71]. These urinary exosomal proteins hold great promise as new diagnostic tools and wait to be further explored.  Table 1 summarizes candidate exosomal protein biomarkers reported to date for diagnostic applications.

### 5.2. Exosomal Nucleic Acids as Diagnostic Biomarkers

Valadi's discovery in 2007 that exosomes contain RNAs [4] has intrigued great interest in the research of exosomal RNAs, especially miRNAs as diagnostic biomarkers. Recent studies have shown that exosomal miRNAs are protected from RNase-dependent degradation and thus can be stably detected in circulating plasma and serum [20, 54, 72], making them “ideal” biomarkers for clinical diagnostic applications.

Exosomal miRNAs have been most frequently exploited as biomarkers for cancer diagnosis. In 2008, a year after Valadi's discovery on exosomal miRNA, Taylor and Gercel-Taylor reported that eight miRNAs previously demonstrated as diagnostic markers for ovarian cancer are found at similar levels in biopsy specimens of ovarian cancer and serum exosomes isolated from the same ovarian cancer patients; however, these exosomal miRNAs could not be detected in normal controls, suggesting that exosomal miRNAs, which are easily attainable, could potentially be used as surrogate diagnostic markers for biopsy profiling [20]. In 2009, Rabinowits and coworkers performed a similar study in lung adenocarcinoma, in which they compared circulating levels of tumor-derived exosomes, exosomal small RNA, and specific exosomal miRNAs between lung adenocarcinoma patients and control subjects. They found similar miRNA patterns in circulating exosomes and tumor biopsies from lung adenocarcinoma patients, both significantly different from those detected in control subjects, suggesting that circulating exosomal miRNA might be useful as a screening test of lung adenocarcinoma [19].

Early detection and diagnosis of prostate cancer may be achieved using the prostate-specific antigen (PSA) test; however, the PSA test suffers from low specificity and a high false-positive rate, which may lead to overtreatment of indolent prostate cancers. Therefore, new markers with a higher diagnostic accuracy are much needed for prostate cancer. In 2008, Mitchell et al. reported that the level of circulating miR-141 is a robust diagnostic marker for prostate cancer [54]. Furthermore, later work by Brase and coworkers showed that serum levels of miR-141 and miR-375 are correlated with tumor progression in prostate cancer [55]. Given that exosomal miRNAs are a major component of RNase-resistant miRNAs in serum or plasma specimens [54, 72], it is plausible to speculate that circulating exosomal miR-141 and miR-375 may be valuable markers for prostate cancer diagnosis.

Exosomal miRNAs also show potential as biomarkers for the diagnosis of esophageal squamous cell cancer (ESCC). In 2013, Tanaka et al. reported that the exosomal miR-21 level is elevated in serum from patients with ESCC versus serum from patients who have benign tumors without systemic inflammation. In addition, exosomal miR-21 is positively correlated with tumor progression and aggressiveness. Importantly, Tanaka et al. claimed that miRNA-21 was not detected in serum that remained after exosome extraction, suggesting that exosomal miRNA-21 is the exclusive source of circulating miRNA-21 [56]. Moreover, Takeshita and coworkers reported in 2013 that serum miRNA-1246 shows a sensitivity of 71.3% and a specificity of 73.9% for ESCC diagnosis. Serum miRNA-1246 is also significantly correlated with the tumor, lymph node, and metastasis stage and has been found to be a strong independent risk factor for poor survival [57]. Interestingly, Takeshita et al. found that although miRNA-1246 is elevated in serum exosomes from ECSS patients, it is not upregulated in ESCC tissue samples, suggesting that serum exosomal miRNA-1246, but not biopsy derived-miRNA-1246, has strong potential as a novel diagnostic and prognostic biomarker in ESCC [57].

Exosomal miRNAs also demonstrate potential as diagnostic biomarkers for cardiovascular diseases and renal fibrosis [59, 60, 63, 64]. Besides, a few studies have shown that, in addition to miRNAs, exosomal mRNAs may be potentially used as biomarkers in clinical diagnostics [62, 64].  Table 2 summarizes candidate exosomal RNA biomarkers reported to date for diagnostic applications.

### 5.3. Exosomes from Other Biofluids as Diagnostic Biomarkers

There is emerging evidence that bodily fluids other than serum and urine may serve as alternate sources for diagnostic exosomes. For instance, Palanisamy et al. reported in 2010 that human saliva contains hundreds of stable mRNA core transcripts, which may be exploited as a possible resource for disease diagnostics [73]. In 2013, Lau et al. showed that saliva exosomes may provide discriminatory biomarkers for pancreatic cancer [74]. Amniotic fluid is another bodily fluid that has been investigated as a potential source of diagnostic exosomal markers. In 2007, Keller et al. isolated exosomes from amniotic fluid and demonstrated for the first time that fetal exosomes are present in amniotic fluid [75], suggesting that exosomes from amniotic fluid may potentially be used in early prenatal diagnostics. Gilad et al. reported in 2008 that miRNAs associated with the human placenta (miR-526a, -527, -515-5p, and -R521) are detectable in both the serum and amniotic fluid of pregnant women and are correlated with pregnancy stage [76]. Further research towards these directions should broaden the application range of exosomal biomarkers.

## 6. Conclusions

Research on the biology, function, and potential application of exosomes has increased exponentially over the past decade. A significant body of literature has demonstrated that one or perhaps the most important biomedical utility of exosomes is their potential application as biomarkers in clinical diagnostics. Compared with biomarkers detected in conventional specimens such as serum or urine, exosomal biomarkers provide comparable or higher specificity and sensitivity attributed to their excellent stability. Particularly, exosomal biomarkers from easily obtainable biofluids such as saliva would be very suitable for clinical applications. Recent technical advances in exosome isolation not only facilitated exosome research but also made exosomal diagnostics more cost-efficient. In addition to exosomal proteins and RNAs, exosomal lipids have also been shown to have diagnostic potential [77]. The utility of exosomes may be further expanded, since they are found not only in mammalian cells but also in diverse pathological microorganisms such as gram-negative bacteria, eukaryotic parasites of the kinetoplast lineage, and opportunistic fungal pathogens [78]. In general, exosomal biomarkers are still in the early discovery/development stage and their potential value in clinical diagnostics waits to be fully explored.

## Figures and Tables

**Table 1:** Summary of exosomal proteins for clinical diagnostic applications.

Biofluid	Disease	Associated proteins	Reference
Plasma	Chronic hepatitis C	CD81	[30]
	Melanoma	CD63, caveolin-1, TYRP2, VLA-4, HSP70, HSP90	[31, 32]
	Glioblastoma	Epidermal growth factor receptor VIII	[33]
	Prostate cancer	Survivin	[34]
	Plasma cell dyscrasias	c-src	[35]

Urine	Acute kidney injury	Fetuin-A, ATF 3	[36, 37]
	Liver injury	CD26, CD81, S1c3A1, CD10	[38]
	Bartter syndrome type 1	NKCC2	[39]
	Bladder cancer	EGF, *α* subunit of Gs, resisitin, retinoic acid-induced protein 3, and so forth.	[40]
	Prostate cancer	PSA, PCA3	[41]

Plasma, cell culture medium, and ascites	Human ovarian cancer	L1CAM, CD24, ADAM10, EMMPRIN, claudin	[42, 43]

**Table 2:** Summary of exosomal RNAs for clinical diagnostic applications.

Biofluid	Disease	Associated RNAs	Reference
Plasma	Ovarian cancer	miR-21, -141, -200a, -200b, -200c, -203, -205, -214	[20]
	Lung cancer	miR-17, -3p, -21, -20b, -223, -301, let-7f	[19, 53]
	Prostate cancer	miR-141, miR-375	[54, 55]
	Esophageal squamous cell cancer (ESCC)	miR-21, miR-1246	[56, 57]
	Breast cancer	miR-21	[58]
	Cardiovascular disease	miR-1, miR-133a	[59, 60]

Cell culture medium	Gastric cancer	Let-7 family miRNAs	[61]
	Colorectal cancer	mRNAs	[62]

Urine	Renal fibrosis	miR-29c, CD2APmRNA	[63, 64]

## Abbreviations

AD: Alzheimer's disease
AKI: Acute kidney injury
DCs: Dendritic cells
EGFRvIII: Epidermal growth factor receptor vIII
ESCC: Esophageal squamous cell cancer
miRNAs: MicroRNAs
MVB: Multivesicular body
PSA: Prostate-specific antigen.
